# *Camellia guiliangii* (Theaceae), a new yellow-flowered *Camellia* species from limestone mountains of southern Yunnan, China

**DOI:** 10.3897/phytokeys.277.198822

**Published:** 2026-07-10

**Authors:** Rujing Zhang, Qingqiu Qin, Guisheng Zhang, Deming He, Jinguo Zhang, Shaoqing Tang, Sujuan Wei

**Affiliations:** 1 Key Laboratory of Ecology of Rare and Endangered Species and Environmental Protection, Ministry of Education, Guangxi Normal University, Guilin 541004, Guangxi, China Key Laboratory of Ecology of Rare and Endangered Species and Environmental Protection, Ministry of Education, Guangxi Normal University Guilin China; 2 Hekou Branch of Managent and Protection Bureau of Daweishan National Nature Reserve, Hekou 661399, Yunnan, China Hekou Branch of Managent and Protection Bureau of Daweishan National Nature Reserve Hekou China; 3 Administration Bureau of Yunnan Wenshan National Nature Reserve, Wenshan 663000, Yunnan, China Administration Bureau of Yunnan Wenshan National Nature Reserve Wenshan China; 4 Management Bureau of Gulinqing Provincial Nature Reserve, Maguan 663700, Yunnan, China Management Bureau of Gulinqing Provincial Nature Reserve Maguan China

**Keywords:** Morphology, phylogeny, single-copy nuclear gene, taxonomy, yellow camellias

## Abstract

*Camellia guiliangii*, a new species of yellow camellia from the limestone mountains of southern Yunnan, China, is described and illustrated. This new species is morphologically similar to *C. fascicularis* but can be clearly distinguished by its connate style, oblate fruit, and seeds densely covered with short, yellowish-brown pubescence. Molecular evidence further confirmed its status as a distinct species, forming a clade that is not closely related to any other yellow camellias. Based on its current distribution and population status, this species should be classified as Critically Endangered (CR) according to the IUCN Red List Categories and Criteria.

## Introduction

*Camellia* species (Theaceae) with yellow flowers, commonly known as “yellow-flowered *Camellia* species” or simply “yellow camellias,” are found in southern China and Vietnam. To date, nearly 50 yellow-flowered *Camellia* species have been reported in Vietnam, and new species continue to be described regularly (Le and Luong 2016; Le et al. 2020; Hoang et al. 2022; Trinh et al. 2023; Quach et al. 2024). In China, the number of yellow camellias is estimated to be 21–24 species (Zhang et al. 2022; Wei et al. 2023; Liu et al. 2025). Most of them are narrowly distributed in Guangxi, with only a few found in Yunnan and Guizhou. All yellow *Camellia* species in China are currently listed as Grade-II National Protected Wild Plants (National Forestry and Grassland Administration 2021).

Yellow camellias are typically distributed across elevations ranging from 125 to 1,600 m. *Camellia inusitata* and *C. bidoupensis* occur at 1,600 m in Bidoup-Nui Ba National Park, Vietnam (Orel et al. 2012; Truong et al. 2022). In China, *Camellia mingii* was previously recorded at the highest elevation, 1,300 m. During field investigations in Hekou Yao Autonomous County, Yunnan, from 2020 to 2025, a unique yellow *Camellia* species was discovered at an elevation of 1,650 m. This plant is characterized by a connate style and seeds covered with short, white pubescence. Further morphological examination and phylogenetic analysis confirmed that this species is new. It is described here as *Camellia guiliangii*.

## Materials and methods

### Observation and comparison of morphological characters

The morphology of the new species was assessed through field observations of living plants and examination of specimens. Voucher specimens were deposited in the Herbarium of the Guangxi Institute of Botany (IBK). For morphological comparisons, relevant literature from both China and Vietnam was consulted. Given its morphological resemblance to *Camellia fascicularis*, detailed morphological comparisons were subsequently made based on specimens from IBSC, IBK, and KUN (accessed via https://www.cvh.ac.cn/) and fresh material. The description of the new species is based on examinations of fresh material, field photographs, and herbarium specimens.

### Taxon sampling and sequencing

A total of 20 yellow-flowered *Camellia* species were included in this study, and *C. hiepii* from sect. *Piquetia* was selected as the outgroup. Detailed information regarding collection localities and voucher specimens is available in the Suppl. material 1. Fresh leaf tissues from 11 species were collected and immediately transported on dry ice to the sequencing facility, where they were subjected to RNA extraction, library construction, and sequencing. At least 6 GB of clean data was generated from each sample. Additionally, resequencing data for the remaining 10 species were obtained from DNA extracted from fresh leaves dried with silica gel. Libraries were constructed and sequenced on the BGI DNBSEQ-Tx platform, yielding approximately 60 Gb of clean data per sample.

### Phylogenetic analyses

*De novo* transcriptome assembly was carried out with Trinity v2.15.1 (Haas et al. 2013). Redundant transcripts were eliminated using CD-HIT v4.8.1 (Li and Godzik 2006). TransDecoder (https://transdecoder.sourceforge.net/) was used to predict coding sequences (CDSs). Reference sets of single-copy orthologous genes (SOGs) were generated using OrthoFinder (Emms and Kelly 2019). After the removal of possible paralogous homologous genes, single-copy nuclear genes (SCNs) were assembled using HybPiper (Johnson et al. 2016). Each SCN was aligned using MAFFT v7.526 (Katoh and Standley 2013) and further trimmed with Gblocks v0.91 (Castresana 2000). Maximum likelihood (ML) analysis was performed using IQ-TREE v2.4.0 (Minh et al. 2020) with 1,000 ultrafast bootstrap replicates.

## Results and discussion

**Figure 1.**
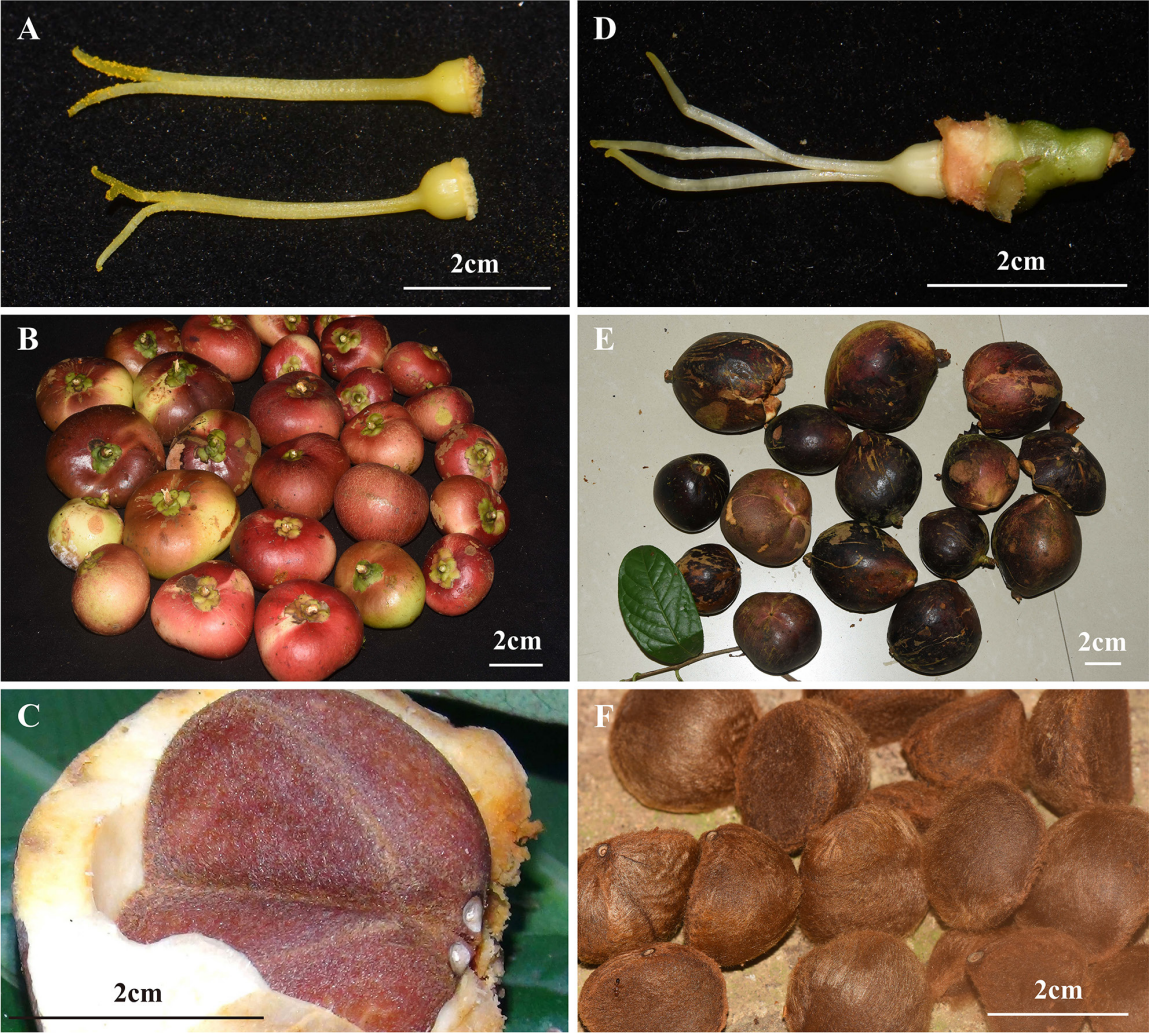
Morphological comparison between *Camellia guiliangii* (**A–C**) and *C. fascicularis* (**D–F**). **A, D**. Style morphology; **B, E**. Fruit shape; **C, F**. Seed indumentum. Photographs by Guiliang Zhang.

Morphological comparison between the newly identified yellow *Camellia* species and *C. fascicularis* reveals three key differences: the new species has connate styles, whereas *C. fascicularis* has distinct styles. Additionally, the fruit of the new species is oblate, whereas that of *C. fascicularis* is globose. The seeds of the new species are densely covered with short, yellowish-brown pubescence, whereas those of *C. fascicularis* are densely covered with long, yellow pubescence (Fig. 1). A detailed morphological comparison between the two species is provided in Table 1.

**Table 1.** Morphological comparison of *C. guiliangii* with *C. fascicularis*.

	***C. guiliangii***	***C. fascicularis***
Leaf	Blade elliptic to oblong, 11–18 × 4–8 cm, secondary veins 9–11, margin serrulate, the teeth dense and sharp on the distal half of the leaf, apex acuminate to caudate	Blade elliptic to oblong-elliptic, 10–19.5 × 5–9.5 cm, secondary veins 9–10, margin sparsely serrate, apex abruptly shortly caudate to caudate
Petiole	1.2–1.8 cm	1.0–1.5 cm
Pedicel length	0.5–0.8 cm	0.6–0.8 cm
Sepals	Outside glabrous, inside white pubescent	Outside sparsely puberulent to subglabrous, inside white pubescent
Petals	Petals 7–9, outer two petals suborbicular, inside white puberulent	Petals 7–8, outer two petals suborbicular, inside white puberulent
Stamens	2.0 cm long, outer filament whorl basally connate for 5 mm	1.8 cm long, outer filament whorl basally connate for 5 mm
Ovary	Globose, glabrous	Ovoid, glabrous
Style	2.8–3 cm long, style connate	2.0 cm long, style distinct
Fruit	Oblate	Globose
Seed	Densely covered with short, yellowish-brown pubescence	Densely covered with long, yellow pubescence

**Figure 2.**
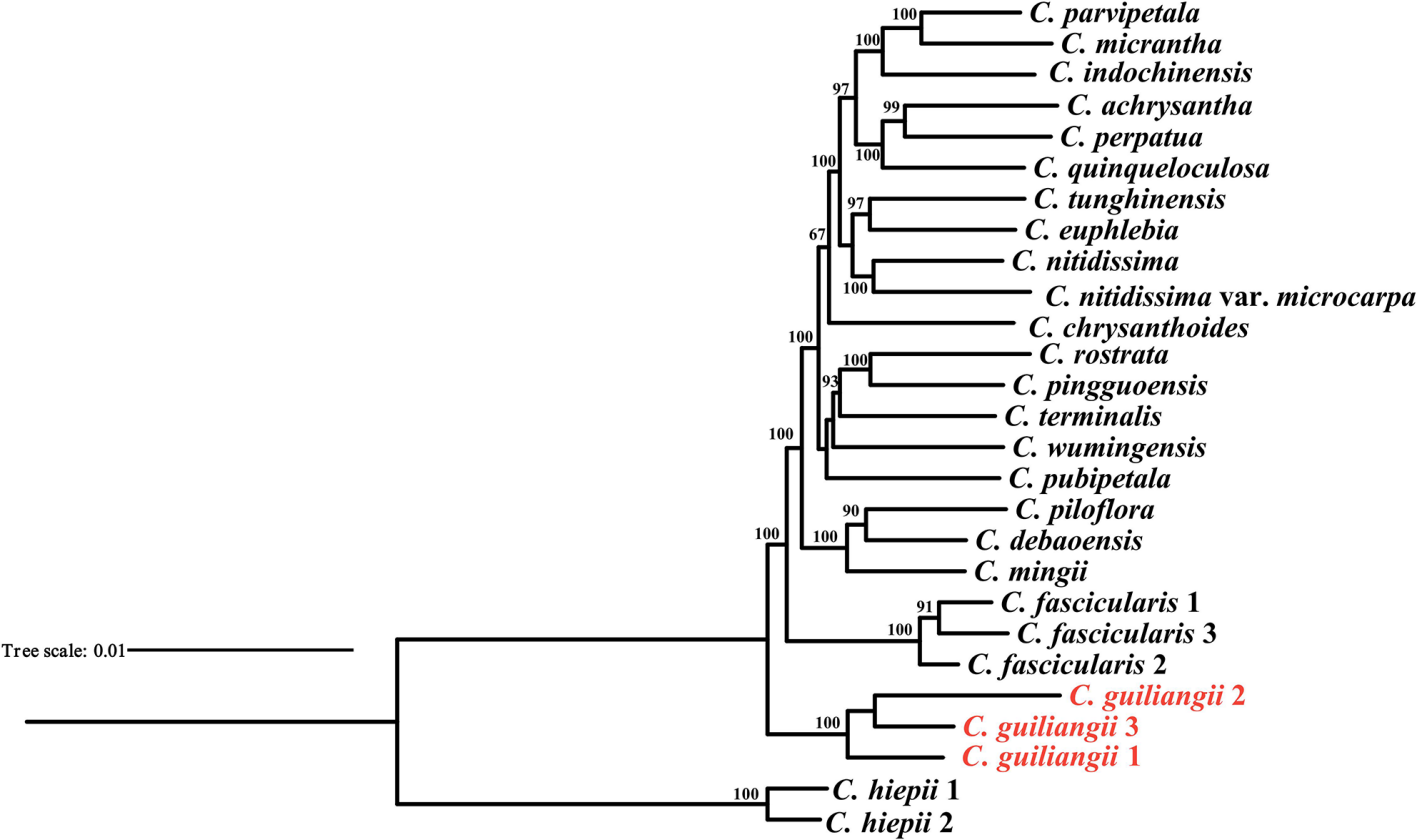
Phylogenetic tree of yellow camellias based on 881 SCNs, with bootstrap support values from ML analyses presented above the branches. The newly described species is highlighted in red.

A total of 881 SCNs were obtained, and the concatenated sequences are available in Suppl. material 2 as a FASTA file. The ML analysis of the 881 SCNs resulted in a well-supported phylogenetic tree, with most nodes receiving full support (100% bootstrap support; Fig. 2). The inferred phylogenetic relationships among species are consistent with the results of a previous phylogenomic study of yellow camellias (Wei et al. 2023). Notably, the new species forms a distinct clade and is not closely related to or nested within any other species (Fig. 2).

### Taxonomic treatment

#### Camellia guiliangii

Taxon classification

Plantae

EricalesTheaceae

Su J.Wei & Shao Q.Tang sp. nov.

93CF6BE2-5949-5B94-BF7B-2E7D16CFC35E

urn:lsid:ipni.org:names:77383599-1

Figs 1, 3

##### Chinese name.

Guì liáng jīn huā chá (贵良金花茶).

**Figure 3.**
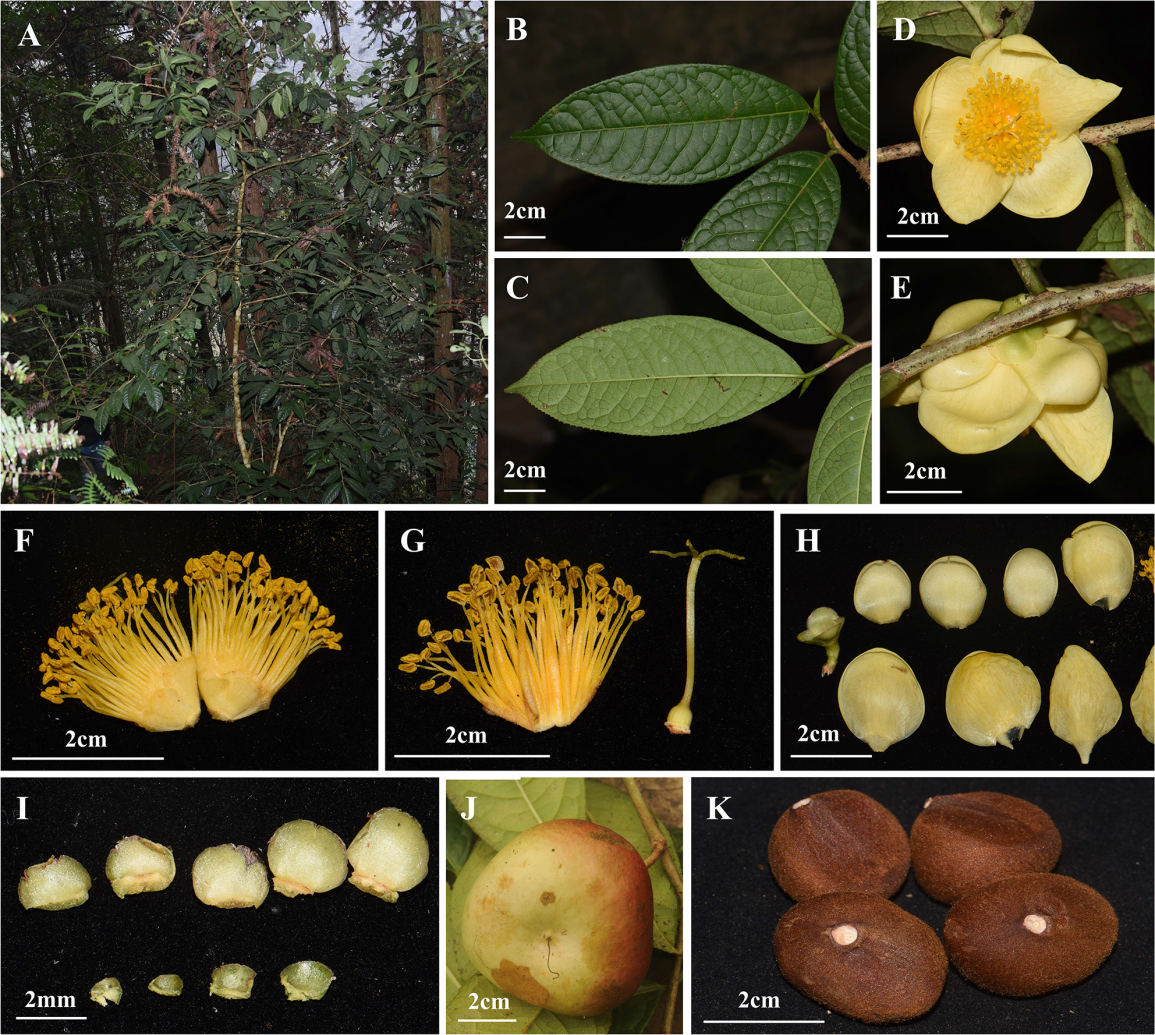
Morphological characteristics of *Camellia guiliangii* sp. nov. **A**. Habit; **B**. Adaxial leaves; **C**. Abaxial leaves; **D**. Front view of flower; **E**. Reverse view of flower; **F**. Outer stamens; **G**. Inner stamens; **H**. Petals; **I**. Bracteoles and sepals; **J**. Fruit; **K**. Seeds. Photographs by Guiliang Zhang.

##### Diagnosis.

*Camellia guiliangii* is morphologically similar to *C. fascicularis* but differs in its connate styles (vs. styles free from the base); oblate fruits (vs. globose); seeds covered with short, yellowish-brown pubescence (vs. densely yellow long pubescence).

##### Type.

China • Yunnan Province: Miechang town, Maguan County, Wenshan Zhuang and Miao Autonomous Prefecture, 22°56'24.43"N, 103°58'51.68"E, alt. 1,650 m, April 2025, *G. L. Zhang zhangGL2025041* (holotype: IBK00473469; isotype: IBK00473470).

##### Description.

***Shrubs*** to small trees, 2–4 m tall. ***Young branches*** yellowish brown or gray-white, glabrous; terminal buds glabrous. ***Leaves*** alternate; petiole 1.2–1.8 cm long, glabrous; leaf blade elliptic to oblong, 11–18 × 4–8 cm, leathery, abaxially pale green, dark or brown glandular punctate, adaxially dark green, both surfaces glabrous, midvein abaxially elevated and adaxially channeled, secondary veins 9–11 on each side of midvein, abaxially raised, and adaxially impressed, base cuneate to broadly cuneate, margin serrulate, the teeth dense and sharp on the distal half of the leaf, apex acuminate to caudate. ***Flowers*** subterminal or axillary, solitary or paired, 3–5 cm in diam. ***Pedicel*** 0.5–0.8 cm, apically thickened; bracteoles four or five, not covering pedicel, green to yellowish green, unequal, 0.2 × 0.2–0.4 × 0.5 cm, semiorbicular, outside glabrous, inside white pubescent, margin ciliolate. ***Sepals*** five, leathery, ovate to semiorbicular, 0.6–0.8 × 0.6–0.8 mm, outside glabrous inside white pubescent, margin ciliolate. ***Petals*** 7–9, pale yellow, in three whorls, outer two petals suborbicular, 1.2–1.5 × 1.3–1.5 cm, concave, outside glabrous, inside white puberulent, inner petals elliptic to oblong-elliptic, 2–3 × 2–3 cm, glabrous, basally connate for 2–5 mm. ***Stamens*** numerous, ca. 2 cm, glabrous, outer filaments whorl basally connate for ca. 5 mm, inner filaments distinct. ***Gynoecium*** 2.8–3 cm long, ovary globose, ca. 3 mm, 3-loculed, glabrous; styles connate into a column ca. 1.8 cm, apically dividing into three arms of ca. 0.5 cm. ***Capsule*** oblate, redwishgreen, ca. 3–6 cm in diam, glabrous, with two seeds per locule; pericarp ca. 3–5 mm thick when dry, splitting into three valves. ***Seeds*** brown, hemispherical, densely pubescent.

##### Etymology.

The species is named in honor of Mr. Guiliang Zhang, who is from the Daweishan National Nature Reserve, Hekou, Yunnan, for his contribution to the investigation and conservation of plant diversity in the Daweishan area. He is also one of the discoverers of this new species.

##### Phenology.

Flowering from October to January of the following year, with peak flowering from November to December; fruiting from October to November.

##### Distribution and habitat.

*Camellia guiliangii* grows in the evergreen broad-leaved forests of limestone hills at elevations of 1,430–1,650 m, dominated by *Carya sinensis* Dode (Juglandaceae), *Michelia lacei* W.W.Sm. (Magnoliaceae), *Phoebe macrocarpa* C.Y.Wu (Lauraceae), and *Itoa orientalis* Hemsl. (Salicaceae).

##### Additional specimens examined (paratype).

China • Yunnan Province: Miechang town, Maguan County, Wenshan Zhuang and Miao Autonomous Prefecture, at an elevation of approximately 1,430 m, 3 December 2025, *G. L. Zhang zhangGL2025056* (GXNU).

##### Provisional conservation status.

Critically Endangered (CR) B1ab(iii), B2ab(iii). Fewer than 100 individuals have been recorded in the town of Miechang, with only two populations found within a very limited distribution range. Given this situation, the species is here assessed as Critically Endangered (CR) according to the IUCN Red List Categories and Criteria (IUCN Standards and Petitions Committee 2024).

## Supplementary Material

XML Treatment for Camellia guiliangii

## Additional information

### Artificial Intelligence (AI) use

The authors accept full responsibility for the content of the manuscript, including the disclosure of any use of AI.

Regarding the use of AI in the preparation of this manuscript, the authors declare the following: DeepSeek.

**Used for**: Language, style and writing.

During the preparation of this manuscript, the authors used AI tools solely for language polishing and grammar checking. This assistance was used in the Introduction and Materials and methods sections.

### Author ORCIDs

R. Zhang https://orcid.org/0009-0007-4735-1480

Q. Qin https://orcid.org/0009-0004-1751-9260

G. Zhang https://orcid.org/0000-0002-5842-3319

D. He https://orcid.org/0009-0005-9070-5235

J. Zhang https://orcid.org/0009-0001-2214-8802

S. Tang https://orcid.org/0000-0001-5532-9784

S. Wei https://orcid.org/0000-0002-2285-4723

### Data availability

All data that support the findings of this study are available in the Supplementary material.

## Supplementary materials

Supplementary material 1 Information of samples used for the RNA-seq and resequencing Data type xlsx https://binary.pensoft.net/file/1713148This dataset is made available under the Open Database License (http://opendatacommons.org/licenses/odbl/1.0/). The Open Database License (ODbL) is a license agreement intended to allow users to freely share, modify, and use this Dataset while maintaining this same freedom for others, provided that the original source and author(s) are credited. Rujing Zhang, Qingqiu Qin, Guisheng Zhang, Deming He, Jinguo Zhang, Shaoqing Tang, Sujuan Wei

Supplementary material 2 Concatenated sequences of 881 single copy nuclear genes Data type txt https://binary.pensoft.net/file/1713149This dataset is made available under the Open Database License (http://opendatacommons.org/licenses/odbl/1.0/). The Open Database License (ODbL) is a license agreement intended to allow users to freely share, modify, and use this Dataset while maintaining this same freedom for others, provided that the original source and author(s) are credited. Rujing Zhang, Qingqiu Qin, Guisheng Zhang, Deming He, Jinguo Zhang, Shaoqing Tang, Sujuan Wei
